# The adult large bowel: describing environment morphology for effective biomedical device development

**DOI:** 10.1088/2516-1091/ad6dbf

**Published:** 2024-08-28

**Authors:** Joseph C. Norton, James W. Martin, Conchubhair Winters, Bruno Scaglioni, Keith L. Obstein, Venkataraman Subramanian, Pietro Valdastri

**Affiliations:** 1 School of Electronic and Electrical Engineering, University of Leeds, Leeds, UK.; 2 Leeds Teaching Hospitals NHS Trust, Leeds, UK.; 3 Division of Gastroenterology, Vanderbilt University Medical Center, Nashville, TN, USA.; 4 Department of Mechanical Engineering, Vanderbilt University, Nashville, TN, USA.

## Abstract

An understanding of the biological environment, and in particular the physical morphology, is crucial for those developing medical devices and software applications. It not only informs appropriate design inputs, but provides the opportunity to evaluate outputs via virtual or synthetic models before investing in costly clinical investigations. The large bowel is a pertinent example, having a major demand for effective technological solutions to clinical unmet needs. Despite numerous efforts in this area, there remains a paucity of accurate and reliable data in literature. This work reviews what is available, including both processed datasets and raw medical images, before providing a comprehensive quantitative description of the environment for biomedical engineers in this and related regions of the body. CT images from 75 patients, and a blend of different mathematical and computational methods, are used to calculate and define several crucial metrics, including: a typical adult size (abdominal girth) and abdominal shape, location (or depth) of the bowel inside the abdomen, large bowel length, lumen diameter, flexure number and characteristics, volume and anatomical tortuosity. These metrics are reviewed and defined by both gender and body posture, as well as – wherever possible – being spilt into the various anatomical regions of the large bowel. The resulting data can be used to describe a realistic “average” adult large bowel environment and so drive both design specifications and high fidelity test environments.

## I. INTRODUCTION

Diseases of the gastrointestinal (GI) tract place a major burden on society and create an urgent need for efficacious, well-tolerated and cost-effective methods of early diagnosis and treatment. A particularly pertinent example is large bowel disease, where the current gold standard method used is conventional flexible colonoscopy, with more than 13 million procedures performed in the USA alone each year [1]. Several drawbacks that are linked to the design of conventional devices, including procedure quality, patient acceptance (comfort) and safety [2] have limited its efficacy and created a significant market pressure to develop novel technologies to improve the standard of care. Many have risen to meet this challenge, with a clear trend emerging in robotic endoscopy [6] and its wide range of concepts, from mobile tracked robots [3] to those with “inch-worm” locomotion [4] and magnetic flexible endoscopes [5]. Efforts have also been made to develop software algorithms to improve the diagnostic yield from the camera images on endoscopes [7][8] and navigation performance of mobile devices [9]. Work is also underway to reconstruct the mucosal surface in 3D [10][11][12] with the goal of providing a real-time method of improving the complete visualization of the anatomy – e.g. a “colon visualization index”. Without this, current clinical practice cannot assure it has visualized all of the anatomy and the quality of the exam is therefore limited.

Despite the plethora of innovations to-date, few have successfully penetrated the market with disruptive solutions. The reasons for this are nuanced and multi-faceted; however, a unanimous contributor is the technical challenge arising from operating in such a complex environment. So, effective medical hardware and software products have a commonality: a design process that considers a deep and accurate understanding of the application environment. This is essential to ensure their successful development not only through definition of appropriate design inputs, but also through their effective pre-clinical evaluation. The latter comprises the thorough testing under the indications for use, ideally in an environment that is both readily available (i.e. cost-effective) and representative of the biological environment. Here devices have a particular need to overcome the challenge of navigating a complex, highly variable environment and benefit from both a theoretical understanding of the anatomy and a model (virtual or physical) to evaluate solutions [14]; on the other hand, algorithms, and data-driven approaches in particular, benefit greatly from access to realistic datasets, including virtual models [15], for designing, training and verifying new approaches.

**Fig. 1.**
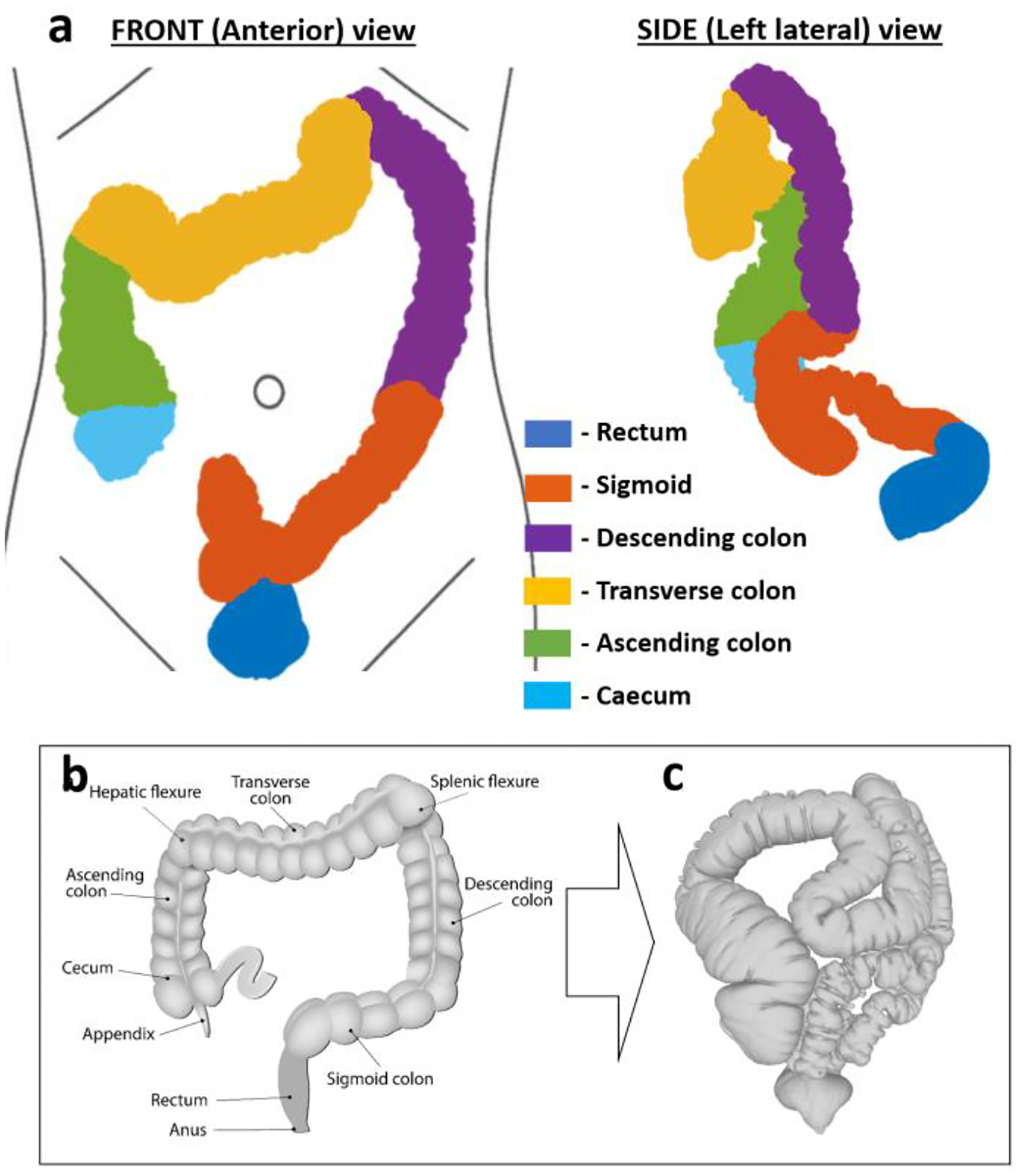
A diagram summarizing the large bowel regions. a. The regions defined on a model of an actual large bowel from CT imaging, b. A typical representation of the large bowel anatomy in literature next to c. An example of a tortuous large bowel.

Considering the size of the market and clear need for it, there is a surprising paucity of detailed literature describing the large bowel environment. The anatomy is usually over simplified, as shown in Fig.1 b.–c.; and, the more realistic information available either lacks robustness due to a small sample number or does not fully describe the environments physical morphology. Furthermore, very few works report the impact of gender and patient abdominal size on the large bowel anatomy, making the application of their data to real-world scenarios difficult.

The goal of this work is to review both literature and raw medical image data to provide a comprehensive overview of the large bowel morphology. This not only provides a valuable resource for driving design inputs of new medical products and their related test environments, but also collects principles and methods to apply to other application areas.

## II. Background

The large bowel (or colon) is the last part of the digestive tract, spanning from the ileocecal valve – the junction between the small intestine and large bowel - and the anus. It serves to extract water and minerals from digested food, and to create and accumulate feces. It is tubular and tortuous in shape, with multiple flexures and regular haustral folds in the tissue giving it a characteristic “sacculated” appearance. It is held loosely in place by mesentery – wide and flat folds of innervated tissue containing lymph and blood vessels. These divide the colon into regions that include: the caecum, ascending colon, transverse colon, descending colon, sigmoid and rectum. They also create two major flexures that are defined by anatomical landmarks, namely: the splenic flexure (near the spleen) and hepatic flexure (near the liver) [13]. Fig.1 provides an overview of these regions.

Numerous methods have been used to describe patient anatomy; however, with the advent of digital medical imaging and image processing software, the scope of analysis dramatically increased. Anatomical structures can be examined in situ and in great detail; the data can also be manipulated to extract specific metrics or physical models can be exported and repurposed. The imaging modality used depends largely on the region of the body and the availability of medical image data. In the GI tract, the most common modality is computed tomography (CT) imaging.

To be able to define useful device specifications and build realistic virtual or synthetic test models, real-world data should be used and processed in a way that is accurate, reliable and accessible. This presents three significant practical challenges: (1) the limited availability of raw medical image data, (2) the high variability inherently found in biological systems (need for high sample numbers) and (3) the effort typically required to manipulate large volumes of complex data. These have constrained previous attempts to completely and accurately describe the large bowel morphology.

Alazmani et al. [16] provide one of the most comprehensive descriptions of the large bowel morphology in literature, in terms of number of metrics reported. However, they report a relatively low sample number given the high variability seen between patients. Furthermore, the patient image datasets were highly selected and may have excluded those with unusual anatomy that still fall within the technically “normal” definition. The methods used to calculate the flexure angle, diameters and volume resulted in limited precision. They also did not describe the flexure characteristics in detail, quantify the lumens location (depth) within the abdomen or the size of the patients’ abdomen.

Khashab et al. [17] provide a compelling work describing length, diameter and number of flexures, and their variation by age, gender and patient size (BMI). However, while they include a very large sample number, they do not explore some key metrics, including volume and flexure details. Furthermore, Prone and Supine patient positions were used interchangeably at the discretion of the examiner which introduces variability as the anatomy is known to alter significantly during this position change.

Zhang [18], Bourgouin [19], Punwani [20], Pritchard [21], Utano [22] and Laframboi et al. [23] all had a very narrow scope of work, focusing on one primary metric. Of these, only Bourgouin, Utano and Pritchard et al. used a high sample number. Zhang and Bourgouin et al. were the only groups exploring the location of the large bowel inside the abdomen: Zhang et al. reporting the absolute minimum depth of the lumen with respect to the abdomen and Bourgouin et al. focused on how key anatomical landmark co-ordinates changed with different patient demographics. Utano et al. reported only length and Punwani et al. looked at how length varied with patient pose. Pritchard et al. explored the volume of the normal and diseased large bowel, measured without lumen distension. Laframboi et al. focused on the flexures found in the large bowel, but only reported the angle from a very small sample number and highlighted the need to include other flexure metrics.

Weber et al. [24] included a high sample number in their work; however, their scope focused only on women who have undergone hysterectomy and the recto-sigmoid morphology. Simplified methods of calculating the metrics were also used. Eickhoff et al. [25] performed a functional assessment of the large bowel anatomy to better understand colonoscopy practice. A large sample number was included; however, several key metrics were not explored, including volume, diameter and flexure details. Furthermore, the level of detail and accuracy of the methods used to calculate metrics were simplified and biased towards examiner opinion. Lastly - and similarly to Eickhoff et al - Hanson et al. [26], while including a high sample number, did not completely describe the anatomy or use accurate methodology to calculate the limited number of metrics reported.

Current literature provides a valuable source of information as a collective, including several clear environmental metrics and useful methods for extracting them. However, it is evident that the majority have not set out with the specific motivation of aiding device or model development, and so the need for a more complete, robust and accurate description of the environment remains. The literature also highlights that in order to accurately describe an environment that is so complex, a high sample number is required and the dataset should have both male and female representation. The metrics should also include key information on the surrounding abdomen – the volume encompassing the large bowel lumen.

This understanding was used to inform the review of publically available adult CT colonography (CTC) image data to provide (1) an accurate description of the environment for product development and (2) methods to perform the same in other anatomical regions.

## III. Quantifying large bowel morphology

A new, more comprehensive review of publically available CTC images was performed to provide a definitive description of the large bowel morphology and a corresponding dataset.

### A. CTC image data source

The Cancer Imaging Archive CT Colonography (ACRIN 6664) dataset [27] consists of abdominal CT scans (DICOM format) of adults indicated for bowel cancer screening, with images taken in both prone and supine positions. Given their indication, this population represents a typical adult population of a wide range of patient sizes and health statuses. Their large bowels were cleaned and inflated with gas prior to the procedure, according to standard clinical practice, to allow it to be clearly imaged. This method also allows the gross morphology to be visualized as normally the bowel is collapsed.

A large sample number of patients from both genders and body positions was used from this repository to ensure a realistic representation of adult anatomy and allow for statistical comparison. The repository was reviewed by sampling patient scans from regular increments throughout the dataset to avoid any biases introduced by the process of collecting and storing them in the original repository. Each patient’s raw CT data was manually inspected and only accepted after passing the following screening criteria:

1. CT images are free from large artefacts or errors.
2. Patient metadata is present (e.g. age and gender).
3. Large bowel is free from large volumes of water/feces (a significant source of processing errors).
4. Large bowel does not have severe lumen narrowing (stenosis; a source of segmentation errors).

**Fig. 2.**
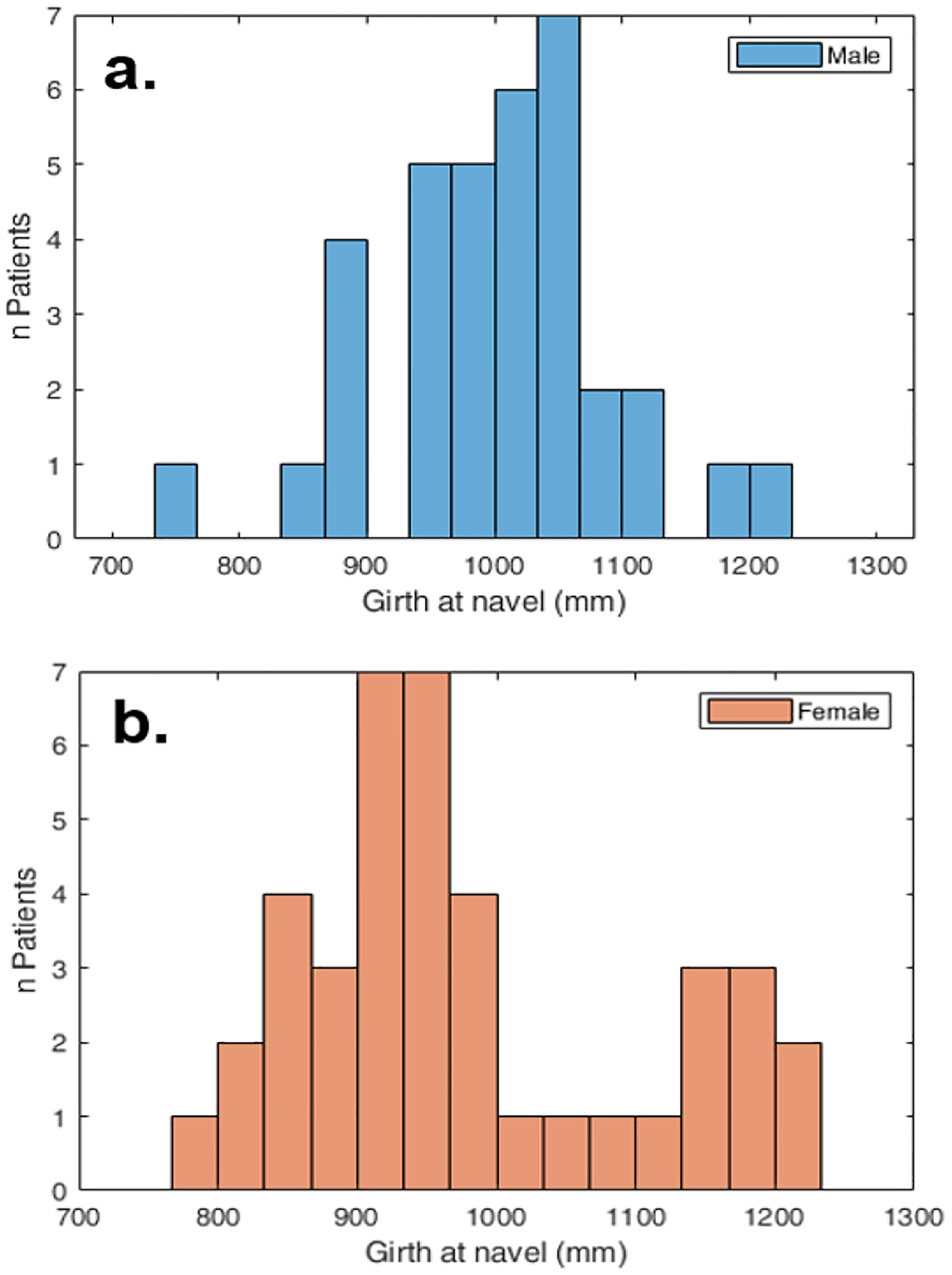
The size of the patient population analyzed in this work. a. Size of the male population, b. Size of the female population.

After screening, a total of 75 patients were chosen and included in this study - 35 male and 40 female; a total that represents a large sample size compared to similar literature. The age range was 50–79 years and the median age was 56 and 55 for males and females respectively. The range of patient sizes is summarized in Fig. 2.

### B. Data formatting

Considering the needs of medical product developers and those wishing to create realistic test environments, the data itself should first be in a format that can be manipulated easily. Table I summarizes the processed dataset produced from the raw CT image data in this study.

From the processed datasets, specific metrics can then be extracted, with the most relevant in this context being summarized in Table II.

Together with the dataset listed in Table I, the patient’s pose (Supine or Prone), gender and age were all recorded. Including both prone and supine datasets, a total of 150 CTC scans were reviewed.

### C. CTC image processing

To improve the accuracy of the outputs, attention was placed on performing thorough numerical methods of characterization that are appropriate for the complexity of the bowel morphology, employing the most effective found in literature or new methods if more appropriate. To begin with, the DICOM files of the screened patients were processed using 3D Slicer (www.slicer.org) [28] and a combination of manual and automated processes to extract the processed dataset, summarized in Table I. This dataset is in a format that facilitates the subsequent metric calculations using MATLAB.

**Fig. 3.**
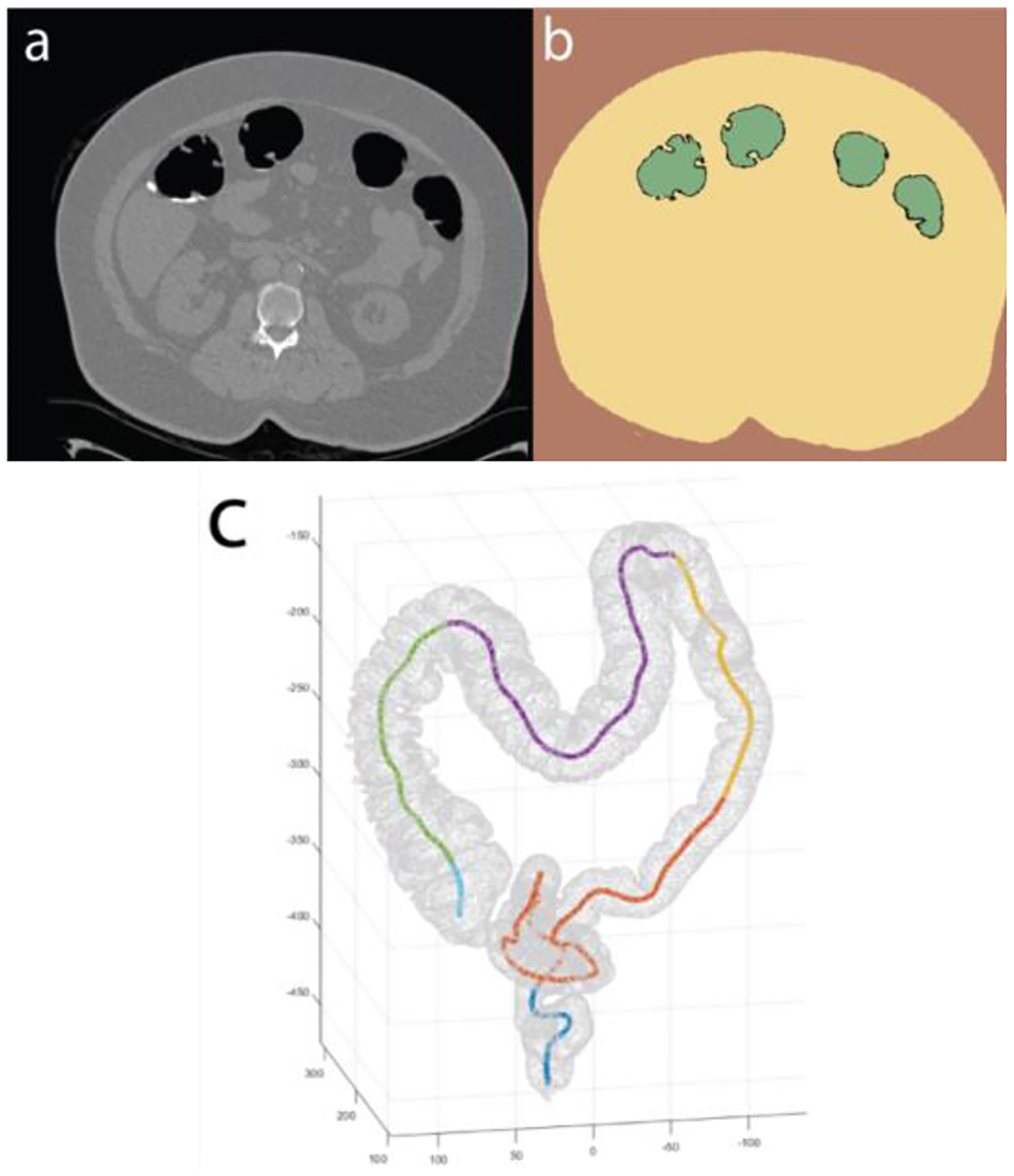
An overview of the DICOM image processing showing a. a raw CT image, b. the segmented and labelled image and c. the 3D model (point cloud) with centerline overlaid.

To segment each DICOM image (Fig. 3.a.), three labels (described in Table I) were first marked manually on 3D Slicer Segment Editor at various points throughout the image set before the three distinct volumes were defined using the “Grow from seeds” function. The output volumes were inspected for errors and manually resolved if required, before being saved as a labelled image set (Fig. 3.b.). The segmentation label of the lumen was then isolated and used to create the 3D model of the large bowel as a dense point cloud (Fig. 3.c.).

The “Extract Skeleton” function was used to determine the center points of the large bowel, represented by a series of approximately 300 fiducial markers with their location registered on the associated DICOM images. These points were visually checked for errors and corrected manually if required. During this process, the main anatomical regions of the large bowel were identified and recorded in the fiducial series by an experienced gastroenterologist. This was done by labelling the fiducial at the junction of each region, including: ‘S’ – Rectum-sigmoid junction; ‘D’ – sigmoid-descending colon junction; ‘T’ – descending-transverse colon junction; ‘A’ – transverse-ascending colon junction and; ‘C’ – ascending-caecum junction. The patient’s navel was also labeled by identifying the fiducial marker on the image slice that represented the center of the navel (a noticeable depression in the abdominal wall). Finally, a smooth centerline of the colon (Fig. 3.c.) was created using the “Mark-ups to model” function which fits a curve to the fiducial series (Moving Least Squares Polynomial, 2nd order, 0.05 Sampling width, and Gaussian weight).

### D. Metric calculation

The following describe the method used to calculate each of the metrics (Table II) from the processed CTC dataset (Table I).

#### Patient abdomen size & shape

**Fig. 4.**
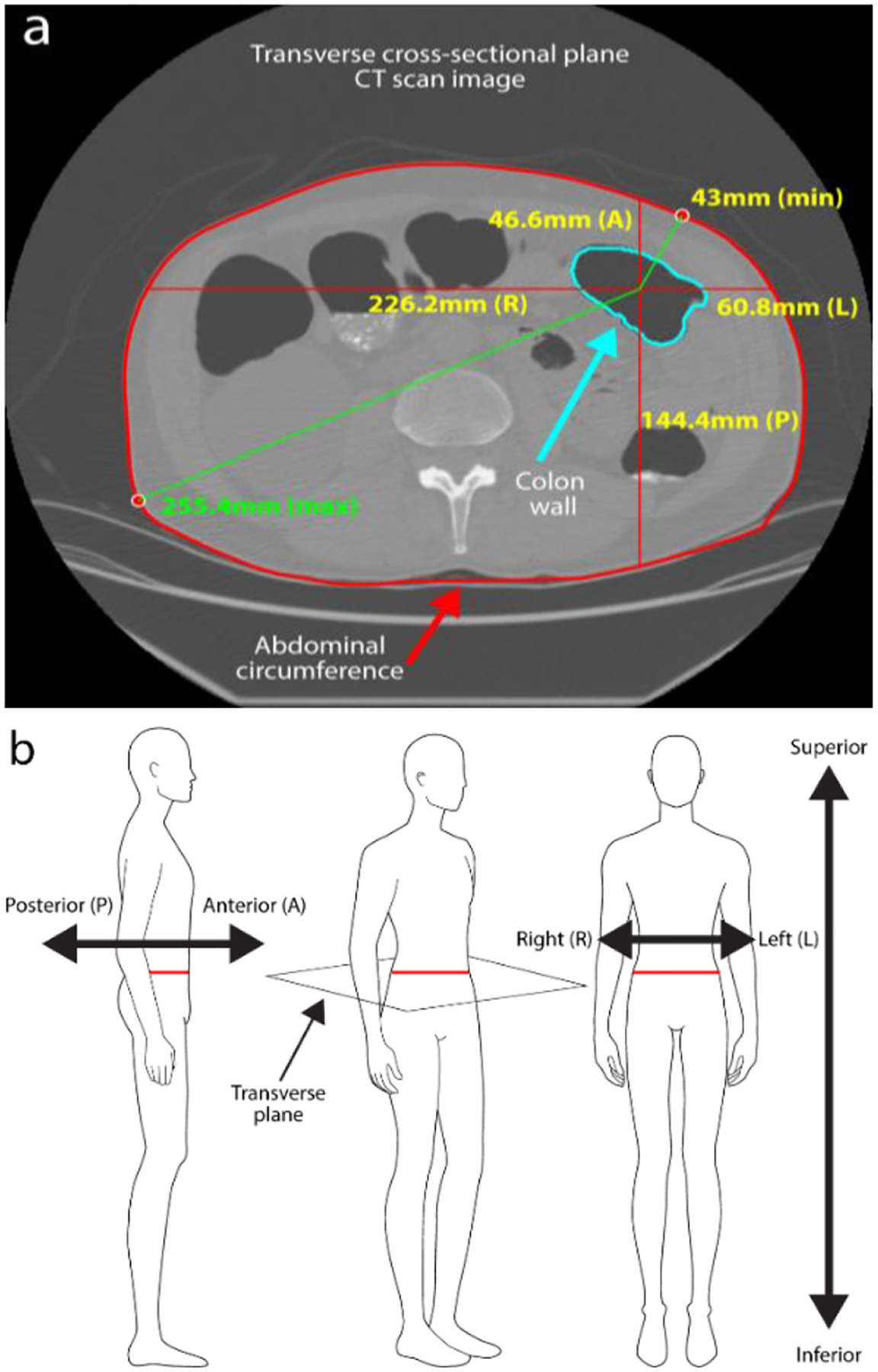
Extracting metrics from the abdominal sections. a. the various metrics defined on each image slice, including perimeter of the abdominal wall and the relative location of the lumen center; b. the definition of patient directions for distance measurements.

The abdominal perimeter was calculated for each DICOM slice by defining the pixels that represent the external edge of the abdomen. To do this a script was written that applies a sliding window (3×3 pixels in size) to the labelled segmented images. Unique pixels that are labelled as “abdomen” and that also neighbor at least one pixel labelled as “air” are stored in an array as points that represent the abdomen. This array then has a convex hull applied to it that fully encompasses the points with appropriate order to accurately capture the shape the abdominal cross-section and precisely define its perimeter (Fig. 4.a.). The output is then multiplied by a scaling factor of the DICOM file to convert the pixel coordinate system to millimeters..

With the coordinates of the perimeter of each abdominal slice defined, the average shape of the abdomen could then be described. The closest simple geometric shape to the abdomen is an ellipse since generally, and especially when lying flat, the human abdominal section is rounded, and wider than it is high (Fig.4.a.). First, the CT image located at the center of the patient’s navel is scanned from left to right to find the maximum vertical height (H). The navel is chosen as it is (a) a landmark that typically represents the apex of the abdomen when the patient is lying flat and (b) is easy to identify and measure in a real-world setting. Maximum horizontal width (W) is measured in a similar fashion but considering all image slices throughout the abdomen. The results from all patients was then averaged to get the H:W ratio of the ellipse that encompasses a typical adult abdomen.

#### Large bowel depth

The position of the bowel within the abdomen is important to understand the operating volume of a product, but is not well reported in literature. So, with the perimeter of each abdominal slice now defined in this new dataset, the distance from the center of the large bowel lumen to this edge was calculated for each image slice. The orthogonal distances (posterior, anterior, left and right directions), as well as the absolute minimum and maximum distances (Fig. 4.a.), were calculated.

#### Length

Length is reported in literature; however, the method used is often of low resolution and is not segmented by bowel region. Here the length of the bowel was calculated as the length of the centerline that was extracted during the DICOM image processing. The labelled fiducial markers then allowed the relative lengths of each anatomical region to be defined.

#### Lumen diameter

**Fig. 5.**
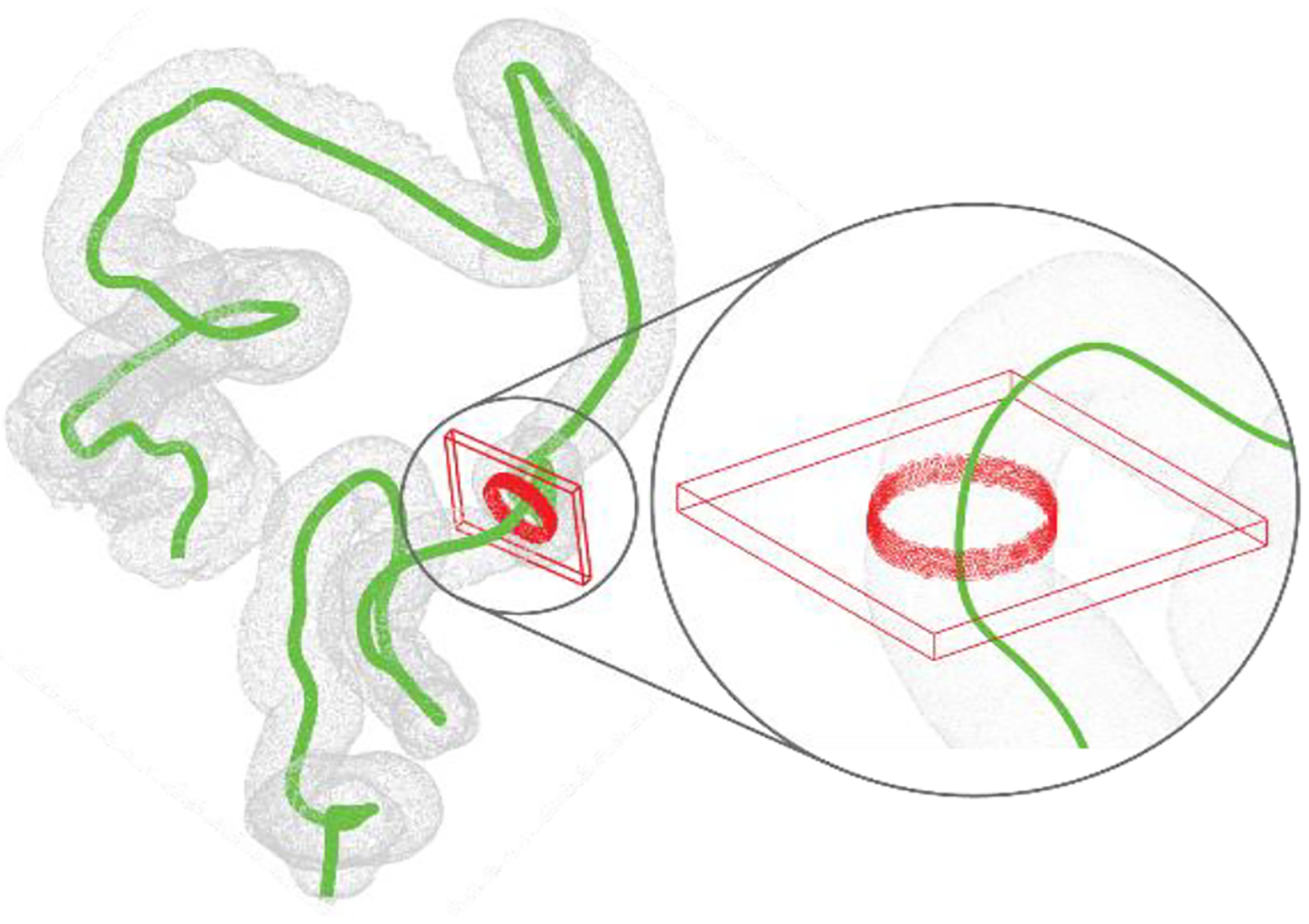
The large bowel lumen is sampled in thin sections orthogonal to the centerline to calculate the diameter.

To calculate the diameter accurately and with high resolution, a new script was written to define the internal perimeter of the lumen. This was done by first moving sequentially along the centerline and defining thin sections of the lumen 3D colon model (Fig. 5).

The points contained within this section are flattened onto the x-y plane to give the cross-sectional shape comprising points with known co-ordinates. Given the complex shape of the bowel and the process of flattening a thin section of bowel that may include a rapid local change in diameter (e.g. at the start of a fold), further processing was required. This involved extracting only the points that define the internal lumen using a search algorithm. Here the points comprising the lumen are stored as a 2D matrix where lumen is assigned a value of 255 and not-lumen is assigned 0. Next, a Moore-neighbor tracing algorithm is used to define points along the internal edge of the lumen using the lumen center as the seed point of the search. Small features in the lumen may cause this output not to conform closely to the lumen and introduces errors in diameter. So a final processing step was performed that involved sequentially moving through the low resolution output of the Moore-neighbor algorithm and finding the nearest point in the surrounding, higher resolution bowel model point cloud. A check is performed to ensure the current selected point is not preceding the previous point and hence a continuous series of points is defined without “back-stepping”. The output is a high resolution cross-section of the internal lumen with known co-ordinates. From here the total area of the cross-section is defined before the diameter is extracted as the diameter of a circle of equivalent area.

#### Volume

With the diameter of the lumen along the entire length of the bowel defined, an accurate volume calculation could be made. The volume of the bowel was first calculated in thin slices and then summed. Given the undulating morphology of the bowel lumen, the most appropriate shape to accurately calculate the volume of these sections is a circular truncated cone with volume described by (1).


(1) $$V=\frac{1}{3}\pi \left(r_{n}^{2}+r_{n}r_{n+1}+r_{n+1}^{2}\right)d$$

Where *r*_*n*_ is the radius of the lumen at the *n*^*th*^ point along the centerline of the bowel and *d* is the distance between two centerline points.

#### Flexure characteristics

The bowel is notoriously tortuous and yet this is often understated, or little information is provided to quantify this. To understand the shape of a typical large bowel, the distinct flexures along the entire bowel centerline must first be identified. To do this, work from Laframboise et al. [23] was used and improved upon.

The circumcenter of every triplet of neighboring points along the centerline is first computed to define a radius of the circumscribed circle. A curvature is then defined as $k_{i}=\frac{\epsilon_{i}}{r}$, where *ϵ*_*i*_ is the unit vector in the direction from the current point to the center of the circumscribed circle with radius *r*. The magnitude of each curvature vector is then computed as $\|k_{i}\|=\sqrt{{k_{i1}}^{2}+{k_{i2}}^{2}+{k_{i3}}^{2}}$. Stepping through ‖*k*_*i*_‖, if the magnitude of this vector is greater than the average magnitude $\left\|\bar{k}\right\|$, then the current point is added into an array that defines the current flexure. Similarly, when the magnitude is less than the average, the flexure is terminated and the algorithm progresses to find the start of the next flexure.

**Fig. 6.**
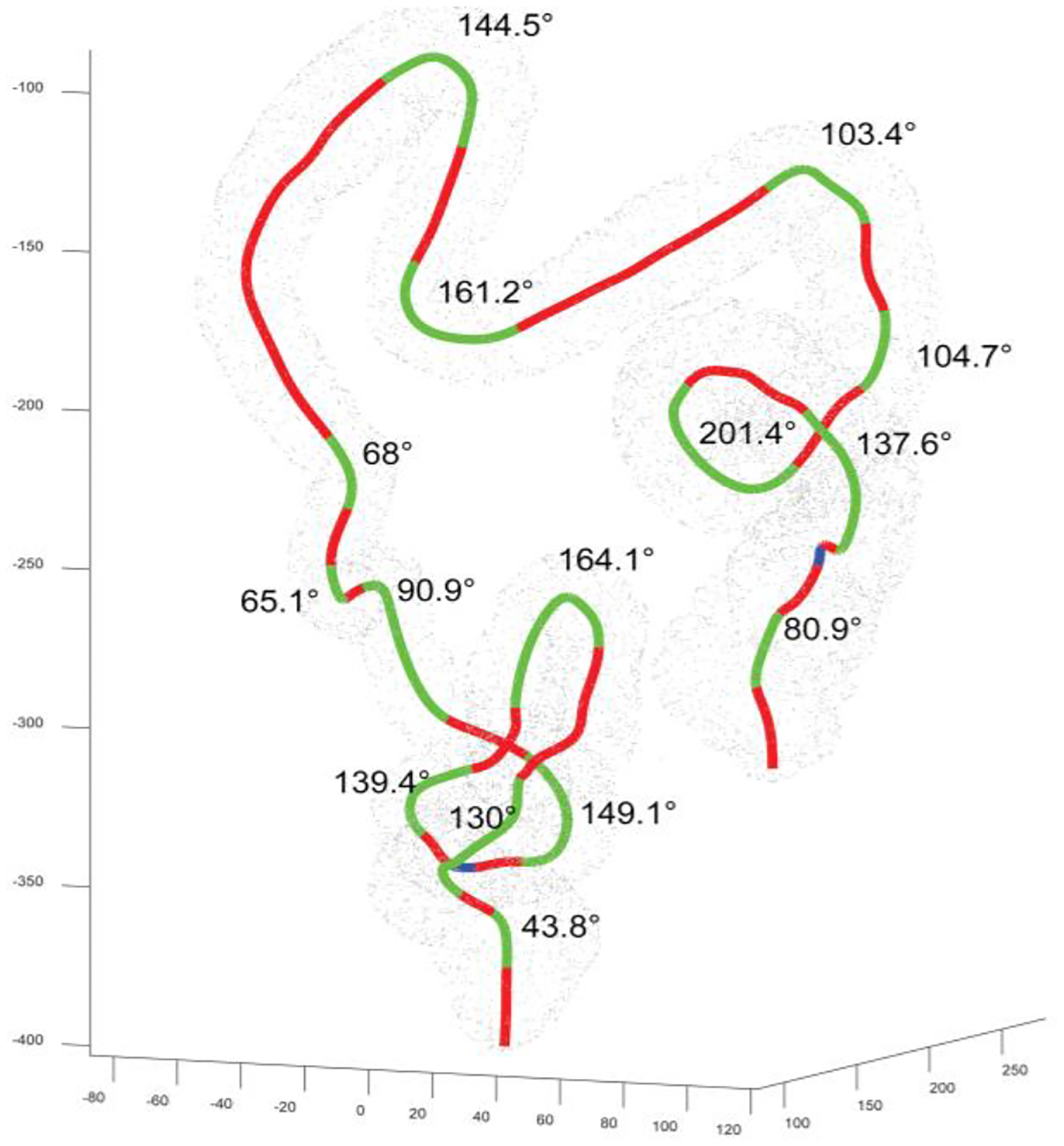
A large bowel centerline with all flexures (green) identified. Red lines indicate no distinct flexure.

Given the complex 3D morphology of the bowel, this method can output 3D curves comprising more than one distinct flexure. A single flexure will tend to have a common heading of normal vectors when defined locally. As the trajectory of the centerline transitions into the next flexure, this common heading goes through a significant change in orientation. Using this characteristic, an algorithm was written that computes a series of unit vectors normal to the centerline and then, using a threshold approach, defines when the change in the angular displacement of the normal vectors exceeds a manually tuned value. This point is marked as the end of the current flexure. Hence, a continuous series of grouped points each forming a distinct flexure can be defined along the entire length of the bowel centerline (Fig. 6).

To compute the angle of these identified flexures, two vectors (*v*_*a*_ and *v*_*b*_) parallel to the centerline were defined as the intersection of the first 3 points and last 3 points of the flexure respectively. A third vector *v*_*c*_ is defined by translating *v*_*b*_ to the origin of *v*_*a*_ before then computing the unit vectors $\hat{v_{a}}=\frac{v_{a}}{\left\|v_{a}\right\|}$ and $\hat{v_{c}}=\frac{v_{c}}{\left\|v_{c}\right\|}$. The angle of the flexure is then represented by (2).


(2) $$\theta =\text{atan}2{\left(\left\|{\hat{v}}_{a}\times {\hat{v}}_{c}\right\|,{\hat{v}}_{a}\cdot {\hat{v}}_{c}\right)}^{2}$$

Flexures with angles < 15◦ or a length less than the average radius of the lumen at that region are discarded, as such slight variations in the centerline when compared to the comparatively large lumen diameter would represent a visually pseudo-straight cylinder.

**Fig. 7.**
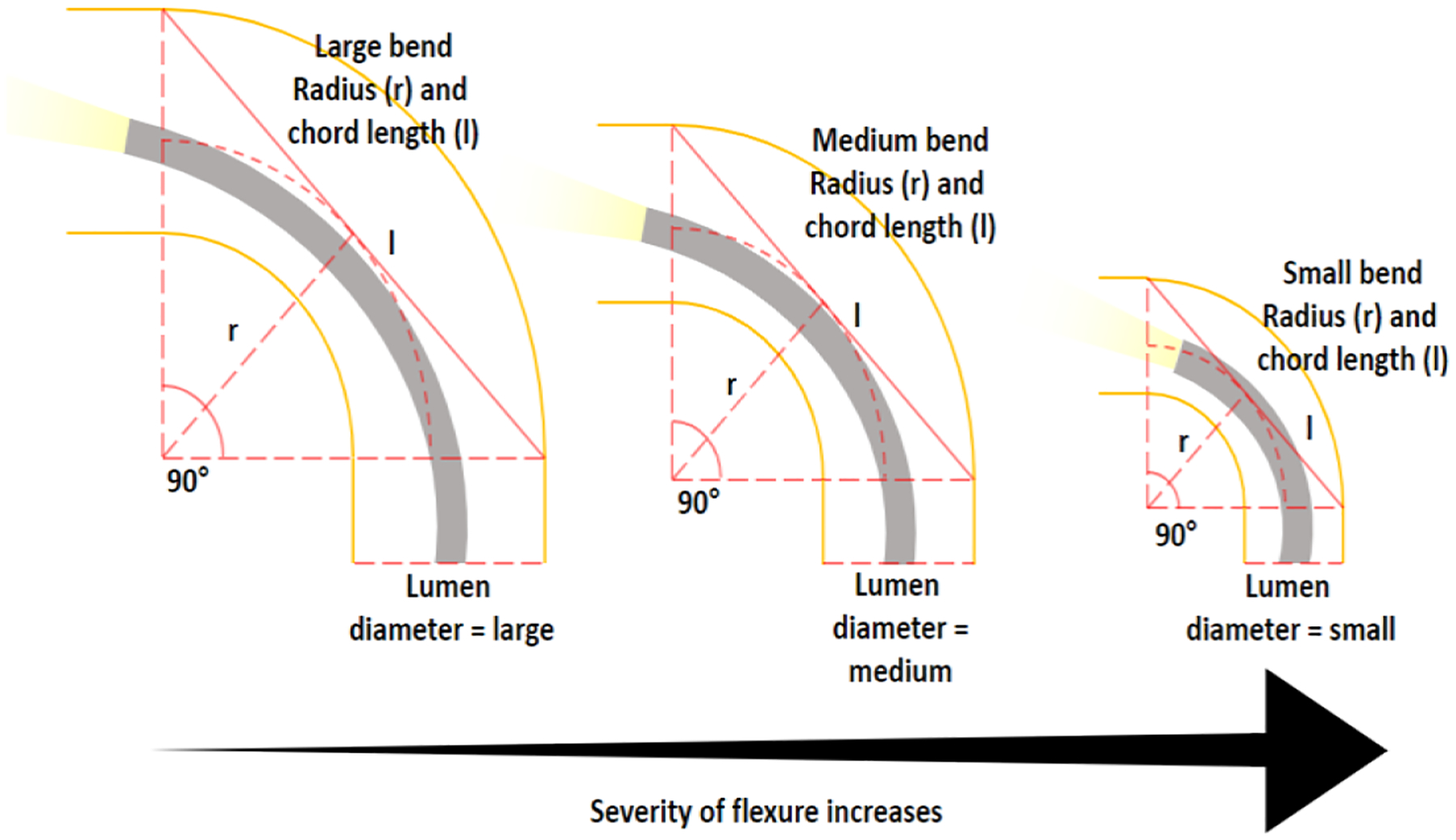
A demonstration of flexure severity being a function of flexure angle, bend radius and lumen diameter.

As shown in Fig. 7, using only the flexure angle is insufficient to quantify how “severe” a flexure is, particularly when considering mobile devices attempting to navigate it. So a customized severity score (S) was used to grade each flexure as a function of not only the flexure angle, but the diameter of lumen around the apex and the flexure’s bend radius. This was normalized according to the average of all the maximum values found in the dataset and simply weighted according to the individual attribute’s intuitive contribution to the severity of a flexure (3).


(3) $$S=3\frac{\theta }{\mu \left(\theta_{max}\right)}+2\frac{\mu \left(r_{min}\right)}{r}+1\frac{\mu \left(d_{min}\right)}{d}$$

This severity scoring provides a useful metric for realistically assessing the tortuosity of the bowel, including a comparison of individual bowels and inter-region differences.

#### Tortuosity

The tortuosity of the bowel - a useful metric to quickly differentiate between different patients and or bowel regions - was calculated by summing the flexure severity scores and dividing by the corresponding length of the large bowel centreline.

## IV. RESULTS: The average adult large bowel

To maintain clarity, the following includes only SUPINE patient position data as this is the most common in clinical practise. For Prone and other more detailed results, refer to the Appendix.

### A. Abdominal shape

When the patient is supine, the average abdominal section shape of an adult female and male can be characterized by an ellipse with height to width ratio of **1:1.40 ± 0.16** and **1:1.37 ± 0.13** respectively. When in prone position, this increases to **1:1.55 ± 0.16** and **1:1.48 ± 0.11** for females and males respectively.

### B. Large bowel length

The insufflated adult large bowel is **164.8 ± 20.4 cm** long in females and **171.7 ± 25.9 cm** in males (Table III). While males have a longer average total length the difference is not statistically significant in this population. On average the rectum and transverse colons are significantly longer in females than in males (P<0.05), while the sigmoid and descending colons are significantly shorter (P<0.05).

### C. Large bowel lumen diameter

The average diameter of the insufflated adult male and female large bowel is **37.2 ± 11.2mm** and **34.9 ± 11.7mm** respectively (Table IV). The female large bowel is smaller on average (p<0.01), with all regions having a statistically significantly smaller diameter, with the exception of the caecum which was similar in size (p>0.5) to the male caecum. The large bowel shows a continuous increase in diameter from recto-sigmoid junction to caecum, with the sigmoid having a diameter of **28.7 ± 7.2 mm** and **26.6 ± 8 mm** for males and females respectively. The caecum has the largest diameter of **53.9 ± 11.5 mm** and **53.7 ± 11.7 mm** for males and females respectively.

### D. Large bowel volume

The average total volume (Table V) of the adult male large bowel is **228.3 ± 67.4 cm**^**3**^. The female anatomy is smaller at **198 ± 66.1 cm**^**3**^ (p<0.05). The transverse colon has a significantly larger volume than all other regions and represents approximately one third of the total volume.

### E. Large bowel flexure characteristics

Tables VI – IX summarise the flexure characteristics in the average adult large bowel. The anatomy is highly tortuous and comprises approximately **15 distinct flexures** along its length, with males having a larger number, but without statistical significance (16.83 vs. 14.87, p>0.05). Males and females have a similar average flexure angle of **111°**. The sigmoid is the most tortuous region of the large bowel having the largest number of severe flexures per length (Table X).

### F. Large bowel depth

Intuitively, the depth of the bowel within the abdomen is proportional to the size of the patient (abdominal perimeter) and is dependent on the direction of measurement. The Appendix provides a detailed overview of the depth of each region of the bowel from all measured directions.

## V. Discussion

The approach of using raw CTC images from a diverse healthy population, a large sample size of both males and females, and both prone and supine positions, all help to provide a realistic average adult large bowel description. The choice of mathematical and computational approaches for processing the data - that maintain the details of the surface morphology (i.e. a high processing resolution) - bolster the quality of the subsequent data analysis. The diverse choice of physical metrics – chosen to supplement and complement those found in literature - support the goal of providing a comprehensive description of the environment. The size and approximate shape of the abdominal section is a useful metric to recreate abdominal models and infer relationships between patient size, shape and large bowel morphology, including its location within the abdominal cavity. For example, the H:W ratio can be used to approximate the width or height of a patient’s abdomen after measuring their abdominal girth. From this, the approximate location of a region of the large bowel can be defined – e.g. the average depth of the descending colon. As expected, when lying in prone position, the abdominal width increases (approximately 8% for males and 11% for females) as the abdomen is compressed.. Combining supine and prone data, and theoretically giving results that most closely fit an ellipse (as the anterior surface is levelled out by the mass of the abdomen), this ratio becomes 1:1.43 ± 0.13 for males and 1:1.47 ± 0.18 for females

Calculating the average diameter of the colon and its individual regions using the perimeter measured at several hundred points along the length gives a more realistic picture of the lumen’s true size. The results clearly highlight the huge variation in human anatomy, e.g. the caecum diameter varies from 20 – 80 mm. This is an important consideration for those developing technologies for this part of the body: while a typical adult anatomy can be defined, a significant portion of the population will vary greatly from this and so design inputs should consider the foreseeable limits.

Interestingly, there was no difference (p>0.05) between the average total length of the male and female large bowel, despite the female anatomy being significantly smaller in volume. This contradicts other literature that reports females having a longer large bowel [17]. The female anatomy has been reported to be more challenging to navigate using a flexible endoscope with various reasons noted [26][25]. This work highlights that one contributor could be a significantly longer (P<0.05) and more tortuous (P<0.05) transverse colon – a region that is also very loosely constrained and so, difficult to traverse with a physical device.

This work provides greater insight into the tortuosity of the large bowel and the specific characteristics of typical flexures, moving beyond the angle and including the bend radius and diameter of the flexure in its description. The severity score and the method of defining the distinct flexures provides the most comprehensive assessment of large bowel tortuosity, defining the average adult tortuosity, as well as the relative tortuosity of each region. A patient specific tortuosity score could aid clinical management of colonoscopy following a CTC scan. Synthetic models (virtual or physical) representing different tortuosity scores could also be used to assist with endoscopist training.

The large bowel can be difficult to access, not only due to the tortuous centreline, but because it spans the entire volume of the abdominal cavity and is near the abdominal wall at some points, and deep in the pelvis in others. This work reports the lumen location with respect to the abdominal wall perimeter in all relevant directions and so allowing realistic localizing of the individual regions from the desired approach direction. The results (Appendix) show a large variation, as expected; however, there is a clear linear trend with depth and abdominal perimeter. Therefore, this work can be used to infer the anticipated depth of the large bowel for a patient of known abdominal perimeter.

This work represents one of the most comprehensive and accurate descriptions of the adult large bowel morphology, but it also highlights several noteworthy limitations that should be considered for future work and when using this (or similar) data to inform critical decisions. The patient demographics are not known in great detail and so any anatomical differences between ethnicities is out of scope. Similarly, the presence of disease and its impact on morphology was not considered when screening the patients’ data. However, given the exclusion of those with severe stenosis through our screening process, the impact on the results is expected to be minimal. Furthermore, the CT image data is a “snap-shot” of the large bowel; while the abdomen, and especially the bowel, is deformable and subject to muscular contracts, and hence temporal morphological variations. Although a standard process was followed during the CTC imaging, variations in the intraluminal pressure used are likely to have a noticeable impact on the large bowel size and in particular the lumen diameter and volume. However, given the mechanical properties and the fact that the bowel wall stiffens sharply when stretched, the variation is not expected to be large. Colonic spasms and other muscular contractions can exaggerate haustral folds and/or produce apparent stenosis that can skew data and produce outliers. The method of calculating diameter and the large sample number gives confidence that this impact is negligible. Standard bowel preparation protocols were followed; however, in several patients there remained a small volume of water and/or faecal matter. Those patients with a large volume of liquid were excluded and those that were included and had some liquid in were manually corrected. However, it is impossible to completely remove all presence of water and faeces from the lumen and this small residual volume will have a slight impact on the diameter and volume metrics. Lastly, the large bowel is a continuous lumen from start to end and hence the precise definition of the anatomical landmarks of the bowel is difficult to achieve. However, the approach of using a single highly trained specialist using current best practice to define the regions will reduce error in examiner variability and give confidence to the technically accurate definition of the regions.

## VI. Conclusion

A review of current literature highlighted a lack of detailed morphological information relating to the large bowel. A new comprehensive review of raw medical CTC images was completed to resolve this. A large sample number, diverse patient population and robust methodology was used to determine a comprehensive set of physical metrics. The abdominal girth and abdominal shape, location (or depth) of the bowel inside the abdomen, large bowel length, lumen diameter, flexure number and characteristics, volume and anatomical tortuosity were all reported for both genders. This provides a significant step forwards in the complete and accurate quantitative description of the large bowel morphology and can be used to inform future medical innovations in this important region of the human body. Noteworthy uses include the definition of device specifications, the design of realistic benchtop simulators for device evaluation and high fidelity virtual models for assessing software algorithms. While focusing on the large bowel, the approach taken in this work can be used to inform evaluations of other biological environments.

## Figures and Tables

**TABLE I:** CTC processed dataset

Patient raw data	Processed dataset	Description
Patient CTC image set (DICOM) n=150	Segmentation label (.nrrd)	The CTC images modified and labelled into three distinct regions: large bowel lumen; the patient’s abdomen (i.e. all tissue and bone surrounding the lumen) and; the air surrounding the patient.
	Labelled fiducial markers (.fcsv)	A series of points with known co-ordinates defining the centerline of the lumen, from anus to caecum. Labelled to identify which points define which anatomical region.
	Large bowel centerline (.ply)	A 3D smooth curve fit to the fiducial markers and visually representing the centerline of the bowel lumen.
	Large bowel 3D model (.ply)	A dense point cloud representing the 3D shape of the large bowel.

**TABLE II:** Metrics generated by this work

Calculated metrics	Description
Abdominal size & shape	The perimeter of the patient’s abdomen, calculated on each CTC image slice and specified at the navel. The shape is described by an ellipse with known Height:Width ratio.
Large bowel depth	The orthogonal distances (Anterior, Posterior, Left and Right) from the centre of the lumen to the surrounding abdominal perimeter, as well as the absolute minimum and maximum distances in any direction. Calculated on each CTC image.
Length	The total length of the large bowel from ano-rectal verge to caecum, and the respective length of each anatomical region.
Diameter	The internal lumen diameter of the large bowel calculated at each fiducial marker along the entire length.
Flexure characteristics	Each distinct flexure along the length of the large bowel identified and described by flexure angle, bend radius and the resulting flexure severity – a function of angle, bend radius and lumen diameter.
Volume	The total volume of the large bowel and the respective volumes of each anatomical region.
Tortuosity	The tortuosity of the large bowel and the respective tortuosity of each anatomical region, defined as a function of the number and severity of flexures per length.

**TABLE III:** The average length of the large bowel (Units = cm)

	Male	Female	P-value
***Rectum***	10.02±2.08	12.65±2.88	**<0.01**
***Sigmoid***	60.61±13.57	53.71±14.67	**<0.05**
***Descending***	25.27±8.42	19.15±3.90	**<0.01**
***Transverse***	49.22±10.79	54.50±11.04	**<0.05**
***Ascending***	19.49±6.05	17.55±6.19	0.18
***Caecum***	7.08±2.27	7.25±2.06	0.74
***TOTAL***	171.69±25.9	164.79±20.41	0.57

**TABLE IV:** The average Diameter of the large bowel (Units = mm)

	Male	Female	P-value
***Rectum***	43.4 ± 12.2	37.8 ± 12.9	**<0.01**
***Sigmoid***	28.7 ± 7.2	26.6 ± 8.0	**<0.01**
***Descending***	35.1 ± 7.0	31.8 ± 7.4	**<0.01**
***Transverse***	41.1 ± 7.0	37.3 ± 8.1	**<0.01**
***Ascending***	48.8 ± 10.7	46.3 ± 11.3	**<0.01**
***Caecum***	53.9 ± 11.5	53.7 ± 11.7	0.59
***TOTAL***	37.2 ± 11.2	34.9 ± 11.7	**<0.01**

**TABLE V:** The average Volume of the large bowel (Units = cm^3^)

	Male	Female	P-value
***Rectum***	22.15 ± 18.52	23.51 ± 17.50	0.86
***Sigmoid***	46.33 ± 23.61	36.16 ± 22.25	0.11
***Descending***	26.89 ± 13.41	16.96 ± 7.42	**<0.01**
***Transverse***	72.43 ± 24.15	67.41 ± 27.31	0.49
***Ascending***	40.47 ± 17.63	33.95 ± 14.42	0.06
***Caecum***	20.60 ± 2.27	10.01 ± 2.06	0.97
***TOTAL***	228.29 ± 67.40	198.01 ± 66.10	**<0.05**

**TABLE VI:** The total number of distinct flexures in the large bowel

	Male	Female	P-value
***Rectum***	0.97	1.28	0.12
***Sigmoid***	7.77	6.36	**<0.01**
***Descending***	2.43	1.56	**<0.01**
***Transverse***	4.34	4.38	0.90
***Ascending***	1.06	1.05	0.98
***Caecum***	0.26	0.23	0.80
***TOTAL***	16.83	14.87	0.20

**TABLE VII:** The average angle of flexures in the large bowel

	Male	Female	P-value
***Rectum***	121.18°	121.24°	1.00
***Sigmoid***	115.39°	112.13°	0.44
***Descending***	103.94°	101.99°	0.78
***Transverse***	107.93°	116.60°	**<0.05**
***Ascending***	94.84°	93.57°	0.86
***Caecum***	100.59°	83.54°	0.30
***TOTAL***	110.63°	111.41°	0.76

**TABLE VIII:** Average bend radius of flexures in the large bowel (Units = mm)

	Male	Female	P-value
***Rectum***	21.80	18.71	0.15
***Sigmoid***	18.61	18.62	0.99
***Descending***	19.16	19.41	0.79
***Transverse***	20.34	20.92	0.33
***Ascending***	22.33	23.64	0.42
***Caecum***	20.19	21.12	0.71
***TOTAL***	19.58	19.78	0.59

**TABLE IX:** Average severity of flexures in the large bowel

	Male	Female	P-value
***Rectum***	3.42	3.48	0.77
***Sigmoid***	3.59	3.59	0.99
***Descending***	3.15	3.25	0.48
***Transverse***	3.10	3.35	**<0.01**
***Ascending***	2.71	2.78	0.65
***Caecum***	2.74	2.38	0.33
***TOTAL***	3.32	3.40	0.17

**TABLE X:** The average tortuosity of the large bowel and its regions

	Male	Female	P-value
***Rectum***	3.78	3.79	0.98
***Sigmoid***	5.28	4.85	0.18
***Descending***	3.45	2.99	0.19
***Transverse***	3.19	3.03	0.44
***Ascending***	1.89	1.80	0.82
***Caecum***	0.97	0.96	0.97
***TOTAL***	3.09	2.90	0.35

**Fig. 8.**
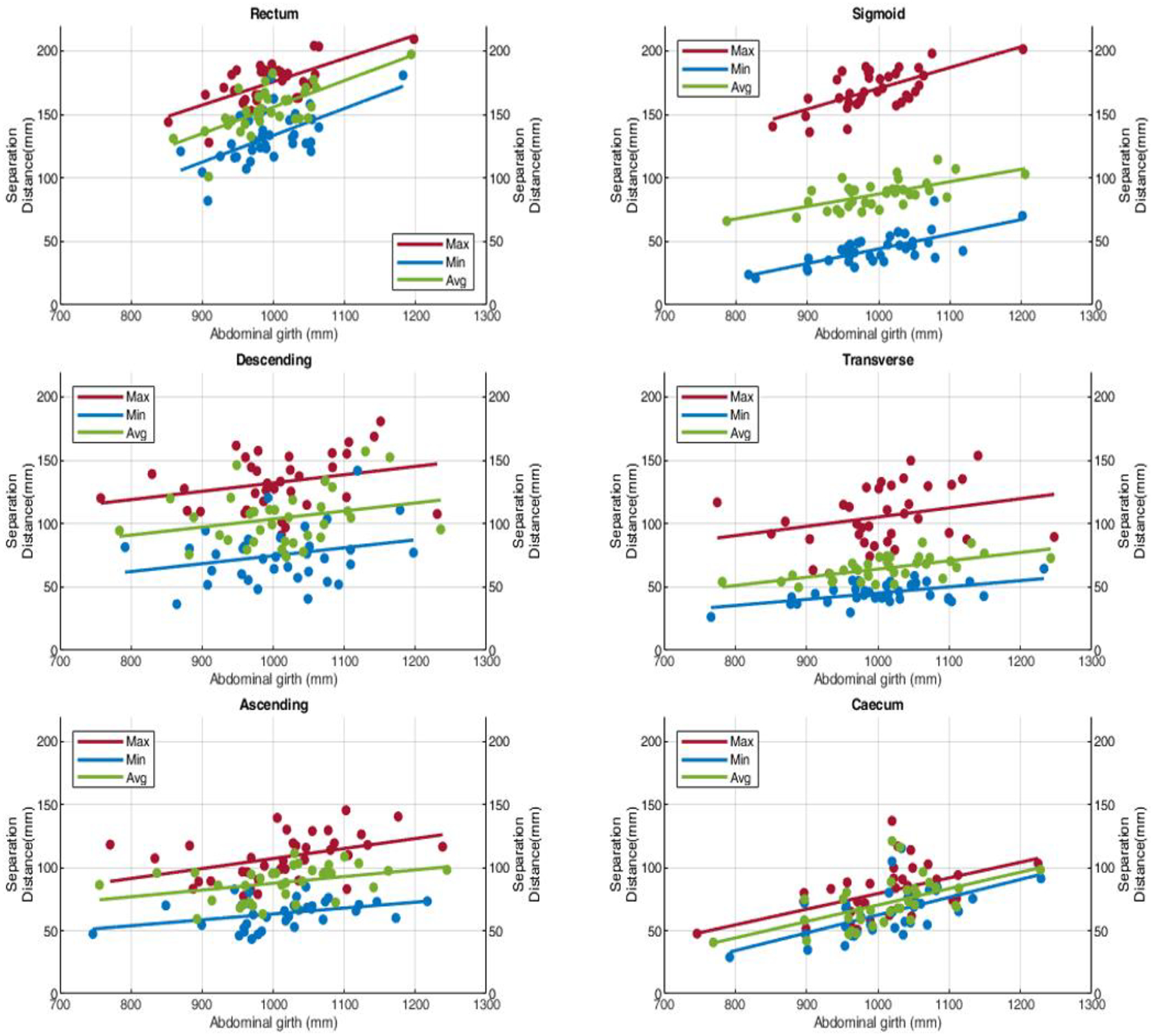
The separation distance (or depth) of the bowel lumen inside the abdominal cavity w.r.t the bowel perimeter. MALE patients, SUPINE body position, ANTERIOR measurement direction.

**Fig. 9.**
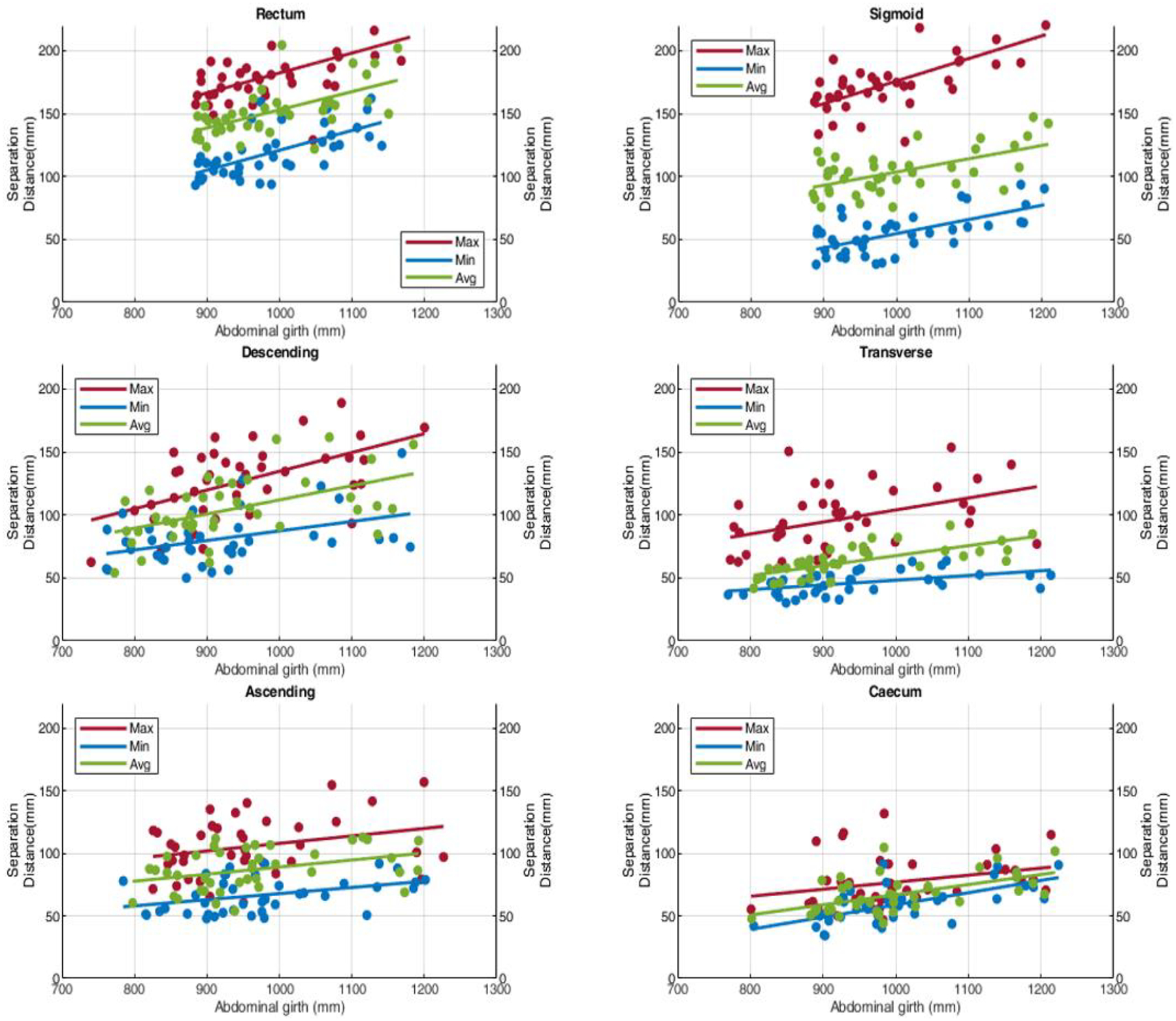
The separation distance (or depth) of the bowel lumen inside the abdominal cavity w.r.t the bowel perimeter. FEMALE patients, SUPINE body position, ANTERIOR measurement direction.

## Data Availability

The data that support the findings of this study are available from the corresponding author upon reasonable request.

## X. Appendix

**TABLE XI:** Average length of the large bowel (Prone; Units = cm)

	Male	Female	P-value
***Rectum***	9.96 ± 1.80	12.86 ± 2.86	**<0.01**
***Sigmoid***	59.68 ± 12.08	53.97 ± 14.39	**0.05**
***Descending***	28.23 ± 9.21	20.96 ± 5.44	**<0.01**
***Transverse***	48.53 ± 11.45	53.79 ± 11.48	**0.05**
***Ascending***	16.25 ± 5.21	15.66 ± 3.51	0.56
***Caecum***	5.90 ± 2.18	6.18 ± 1.95	0.57
***TOTAL***	167.87 ± 25.72	162.67 ± 20.07	0.67

**TABLE XII:** Average Diameter of the large bowel (Prone; Units = mm)

	Male	Female	P-value
***Rectum***	47.9 ± 11.9	42.1 ± 13.3	**<0.01**
***Sigmoid***	27.8 ± 7.7	26.5 ± 7.3	**<0.01**
***Descending***	34.2 ± 7.6	30.9 ± 7.7	**<0.01**
***Transverse***	38.9 ± 7.5	35.5 ± 7.9	**<0.01**
***Ascending***	46.8 ± 11.7	45.1 ± 11.3	**<0.05**
***Caecum***	51.5 ± 11.1	50.1 ± 12.3	0.95
***TOTAL***	35.7 ± 11.2	33.9 ± 11.2	**<0.01**

**TABLE XIII:** Average Volume of the large bowel (Prone; Units = cm^3^)

	Male	Female	P-value
***Rectum***	31.68 ± 18.59	30.03 ± 19.12	0.73
***Sigmoid***	44.04 ± 22.43	36.15 ± 20.62	0.21
***Descending***	28.83 ± 14.59	18.16 ± 7.90	**<0.01**
***Transverse***	64.02 ± 22.75	58.22 ± 23.06	0.30
***Ascending***	33.63 ± 17.35	30.43 ± 13.52	0.40
***Caecum***	16.88 ± 9.68	17.00 ± 11.93	0.67
***TOTAL***	219.09 ± 66.53	190.00 ± 58.23	**<0.05**

**TABLE XIV:** Total number of distinct flexures in the large bowel (Prone)

	Male	Female	P-value
***Rectum***	0.69	1.21	**<0.01**
***Sigmoid***	7.34	6.08	**<0.01**
***Descending***	2.77	2.03	**<0.05**
***Transverse***	3.86	4.28	0.28
***Ascending***	0.86	0.92	0.72
***Caecum***	0.23	0.28	0.60
***TOTAL***	15.74	14.79	0.53

**TABLE XV:** Average angle of flexures in the large bowel (Prone)

	Male	Female	P-value
***Rectum***	127.69°	125.15°	0.80
***Sigmoid***	110.41°	113.58°	0.46
***Descending***	111.07°	102.86°	0.18
***Transverse***	110.49°	111.64°	0.81
***Ascending***	93.46°	103.27°	0.30
***Caecum***	88.81°	92.23°	0.77
***TOTAL***	110.06°	111.44°	0.59

**TABLE XVI:** Average bend radius of flexures in the large bowel (Prone; Units = mm)

	Male	Female	P-value
***Rectum***	20.5	19.0	0.32
***Sigmoid***	19.8	19.6	0.83
***Descending***	19.7	18.4	0.16
***Transverse***	19.7	20.1	0.57
***Ascending***	22.5	20.9	0.37
***Caecum***	20.6	20.5	0.94
***TOTAL***	20.0	19.6	0.50

**TABLE XVII:** Average severity of flexures in the large bowel (Prone)

	Male	Female	P-value
***Rectum***	3.63	3.49	0.50
***Sigmoid***	3.64	3.60	0.70
***escending***	3.39	3.25	0.29
***Transverse***	3.31	3.27	0.67
***Ascending***	2.82	3.11	0.15
***Caecum***	2.67	2.77	0.73
***TOTAL***	3.45	3.40	0.34

**TABLE XVIII:** Average tortuosity of the large bowel (Prone)

	Male	Female	P-value
***Rectum***	2.66	3.52	0.13
***Sigmoid***	4.87	4.65	0.20
***Descending***	3.80	3.55	0.61
***Transverse***	2.96	2.91	0.82
***Ascending***	1.79	2.10	0.45
***Caecum***	0.96	1.38	0.40
***TOTAL***	2.87	3.02	0.48

**TABLE XIX:** The average distances from the lumen center of each region to the abdominal perimeter (*MALE patients; units = cm*)

		Rectum			Sigmoid			Descending			Transverse			Ascending			Cecum		
		Min	Avg	Max	Min	Avg	Max	Min	Avg	Max	Min	Avg	Max	Min	Avg	Max	Min	Avg	Max
Left distance (cm)	Supine	17.69	18.55	19.41	4.73	11.98	20.16	4.58	6.17	8.57	5.58	12.83	23.18	21.47	25.33	27.62	19.38	20.92	22.52
	Prone	16.93	17.99	18.91	5.11	13.15	20.91	4.74	6.44	9.00	5.98	16.25	27.91	25.16	27.55	29.26	23.30	24.67	26.08
	*P-value*	0.05	0.12	0.19	0.25	**0.01**	0.19	0.47	0.27	0.30	0.30	**0.01**	**0.01**	**0.01**	**0.01**	**0.01**	**0.01**	**0.01**	**0.01**
Right distance (cm)	Supine	17.16	18.04	18.94	10.99	18.04	26.58	24.18	27.10	29.31	6.63	16.04	27.10	5.51	6.81	8.56	6.48	7.48	8.69
	Prone	16.51	17.47	18.45	11.94	19.36	27.90	24.86	27.95	30.31	6.60	17.46	28.23	5.59	7.12	8.86	7.02	7.94	8.99
	*P-value*	0.09	0.12	0.21	0.22	**0.02**	0.07	0.31	0.16	0.09	0.94	**0.02**	0.07	0.73	0.32	0.56	0.26	0.41	0.68
Anterior distance (cm)	Supine	13.22	15.43	17.46	4.35	8.71	16.95	7.52	10.42	13.25	4.55	6.47	10.56	6.38	8.81	10.83	6.39	7.17	8.05
	Prone	13.70	15.98	17.69	4.27	9.10	17.05	7.99	11.40	14.75	4.95	7.46	12.04	7.93	10.97	12.70	7.06	7.76	8.53
	*P-value*	0.29	0.18	0.56	0.78	0.18	0.79	0.42	0.08	**0.01**	0.08	**0.00**	**0.02**	**0.00**	**0.00**	**0.00**	0.18	0.24	0.39
Posterior distance (cm)	Supine	7.05	8.58	10.35	7.94	16.39	21.08	10.16	13.10	16.64	13.73	20.21	23.39	12.80	15.41	19.48	16.93	18.10	18.99
	Prone	7.50	9.22	11.47	7.87	14.97	19.64	7.49	10.48	14.59	11.07	16.82	20.39	8.68	10.78	14.27	13.65	14.54	15.30
	*P-value*	0.12	**0.02**	**0.01**	0.83	**0.01**	**0.01**	**0.01**	**0.01**	**0.01**	**0.01**	**0.01**	**0.01**	**0.01**	**0.01**	**0.01**	**0.01**	**0.01**	**0.01**

**TABLE XX:** The average distances from the lumen center of each region to the abdominal perimeter (*FEMALE patients; Units = cm*)

		Rectum			Sigmoid			Descending			Transverse			Ascending			Cecum		
		Min	Avg	Max	Min	Avg	Max	Min	Avg	Max	Min	Avg	Max	Min	Avg	Max	Min	Avg	Max
Left distance (cm)	Supine	16.50	17.84	19.12	6.33	13.21	20.06	4.99	6.46	8.65	5.34	11.29	21.36	19.87	23.35	25.48	18.60	20.11	21.61
	Prone	15.97	17.52	18.88	7.52	14.21	20.62	5.14	6.94	9.35	5.51	13.45	25.11	22.96	24.97	26.45	22.94	24.21	25.51
	*P-value*	0.33	0.50	0.62	**0.01**	0.07	0.39	0.56	0.09	0.07	0.58	**0.01**	**0.01**	**0.01**	**0.01**	0.12	**0.01**	**0.01**	**0.01**
Right distance (cm)	Supine	16.47	17.65	18.86	13.95	19.24	26.03	22.32	24.83	26.61	6.82	15.62	23.92	5.82	7.21	9.09	7.71	8.76	9.95
	Prone	16.06	17.41	18.87	14.37	20.24	26.74	22.81	25.02	27.14	6.75	18.18	26.90	6.10	7.60	9.31	7.50	8.78	9.93
	*P-value*	0.37	0.56	0.99	0.53	**0.04**	0.25	0.44	0.77	0.48	0.87	**0.00**	**0.00**	0.35	0.25	0.66	0.70	0.96	0.98
Anterior distance (cm)	Supine	11.84	15.13	18.21	5.37	10.37	17.65	8.03	10.40	12.74	4.53	6.33	9.81	6.62	8.74	10.64	5.92	6.71	7.65
	Prone	12.70	15.47	17.72	5.30	10.09	16.85	9.21	12.22	14.38	5.17	7.47	11.89	7,52	10.67	12.83	5.93	6.89	7.89
	*P-value*	0.08	0.46	0.32	0.84	0.46	0.10	**0.02**	**0.01**	**0.01**	**0.01**	**0.01**	**0.01**	**0.03**	**0.01**	**0.01**	0.98	0.69	0.67
Posterior distance (cm)	Supine	6.67	8.70	10.72	7.74	14.60	19.26	9.55	11.53	14.06	12.85	17.97	21.13	11.74	14.28	17.81	16.77	18.22	19.17
	Prone	7.19	9.23	11.56	7.84	13.84	18.85	6.51	8.52	12.07	9.10	14.53	18.07	7.99	10.37	14.29	13.95	15.38	16.67
	*P-value*	0.17	0.13	**0.03**	0.78	0.13	0.43	**0.01**	**0.01**	**0.01**	**0.01**	**0.01**	**0.01**	**0.01**	**0.01**	**0.01**	**0.01**	**0.01**	**0.01**
